# The Cognitive Relevance of a Formal Pre-incision Time-out in Surgery

**DOI:** 10.1145/3452853.3452867

**Published:** 2021-04-26

**Authors:** Lauren R. Kennedy-Metz, Roger D. Dias, Marco A. Zenati

**Affiliations:** Medical Robotics and Computer-Assisted Surgery Lab, Harvard Medical School and VA Boston Healthcare System, Boston, MA, US; Human Factors and Cognitive Engineering Lab, STRATUS Center for Medical Simulation, Brigham and Women’s Hospital, Harvard Medical School, Boston, MA, US; Medical Robotics and Computer-Assisted Surgery Lab, Harvard Medical School and VA Boston Healthcare System, Boston, MA, US

**Keywords:** Pre-incision time-out, Cardiac surgery, Mental workload, Situation awareness, Team mental models

## Abstract

Surgical time-outs are designed to promote situation awareness, teamwork, and error prevention. The pre-incision time-out in particular aims to facilitate shared mental models prior to incision. Objective, unbiased measures to confirm its effectiveness are lacking. We hypothesized that providers’ mental workload would reveal team psychophysiological mirroring during a formal, well-executed pre-incision time-out. Heart rate variability was collected during cardiac surgery cases from the surgeon, anesthesiologist, and perfusionist. Data were analyzed for six cases from patient arrival until sternal closure. Annotation of surgical phases was completed according to previously developed standardized process models of aortic valve replacement and coronary artery bypass graft procedures, producing thirteen total surgical phases. Statistical analysis revealed significant main effects. Tukey HSD post hoc tests revealed significant differences across provider roles within various phases, including Anesthesia Induction, Heparinization, Initiation of Bypass, Aortic Clamp and Cardioplegia, Anastomoses or Aortotomy, Separation from Bypass, and Sternal Closure. Despite these observed differences between providers over various surgical phases, the Pre-incision Time-out phase revealed almost negligible differences across roles. This preliminary work supports the utility of the pre-incision safety checklist to focus the attention of surgical team members and promote shared team mental models, measured via psychophysiological mirroring, using an objective mental workload measure. Future studies should investigate the relationship between psychophysiological mirroring among surgical team members and the effectiveness of the pre-incision time-out checklist.

## 1 INTRODUCTION

The inherent complexity associated with healthcare systems, and in particular with surgery, warrants an analytical approach focusing on the scope of cognitive demands through cognitive engineering methods [1]. The cognitive basis of medical error in surgery has a relatively recent history but our understanding of it has advanced notably due to sentinel observations and increasing efforts to interpret and overcome cognitive-based error [2–4]. A notable result of the impact of these observations is the implementation of the World Health Organization’s (WHO) Surgical Safety Checklist [3], which has contributed to enhanced teamwork and communication [5] and improved post-operative patient outcomes [6]. Checklist-guided surgical time-outs have produced a reduction in rates of death and complications [7, 8], and implementation of the pre-incision time-out in particular has been associated with a decrease in medical error [9] and a decline in 120-day mortality [10] in recent studies.

Despite these findings, effectiveness of the pre-incision time-out has yet to be demonstrated via objective measures of mental states, such as mental workload (MWL). Though definitions of MWL often vary, it is generally accepted that individuals have limited cognitive and attentional capacity [11]. MWL is important in the context of surgery due to growing evidence of the influence of cognition and cognitive errors on surgical performance [12, 13], and can be accurately and reliably measured via objective means. Heart rate variability (HRV) is the most extensively utilized measure of workload during surgery [14], and evidence supports its accuracy in reflecting mental workload and procedure workload levels in complex systems [15, 16], including surgery [17].

To date, despite improvements in patient outcomes and perceived team dynamics attributed to surgical time-outs, there is a lack of objective evidence supporting their effectiveness. The aim of this study is to investigate the relationship between providers’ MWL and surgical phases within aortic valve replacement (AVR) and coronary artery bypass graft (CABG) surgeries at a tertiary academic hospital, with special interest in the pre-incision time-out phase. Given recent evidence suggesting that mirrored changes in team workload reflect the presence of shared team-wide mental models [17,18], we hypothesized that providers’ MWL would reveal mirrored team workload states across individuals during a formal, well-executed pre-incision time-out.

## 2 METHODS

The research protocol for this project was approved by the Institutional Review Boards of VA Boston Healthcare System and Harvard Medical School (IRB#3047). All individuals entering the operating room (OR) during recordings provided written informed consent prior to the start of the surgery.

Data were collected during AVR and CABG procedures in the cardiac OR of a tertiary teaching hospital between January 2017 and November 2018. Data sources included two GoPro HERO4 Black Edition cameras (San Mateo, CA) for video capture of 1) the entire OR and 2) the surgical field; three Sony ICD-PX440 audio recorders (Tokyo, Japan) for audio capture of 1) the attending surgeon, 2) the attending anesthesiologist, and 3) the primary perfusionist; and three Polar V800 chest straps with H10 sensors (Kempele, Finland) for heart rate capture of 1) the attending surgeon, 2) the attending anesthesiologist, and 3) the primary perfusionist.

For each case, all audio, video, and physiological recordings were manually time synced and integrated by one researcher (LKM). Data were analyzed from the time the patient was transferred onto the operating table until the end of sternal closure. Identification and annotation of surgical phases was done manually by one coder (LKM), according to previously developed standardized process models of AVR and CABG procedures [19]. This previous elaboration of AVR and CABG process models produced distinct phases of cardiac surgery based on observable process steps; 13 phases within AVR and 14 phases within CABG. For the purpose of analyzing and comparing complete data sets, the Vessel Harvesting phase of CABG was excluded, leaving the following phases common to both AVR and CABG procedures: 1) Pre-induction, 2) Anesthesia Induction, 3) Sterile Prepping, 4) Pre-incision Time-out, 5) Sternotomy, 6) Heparinization, 7) Aortic Cannulation, 8) Initiation of Bypass, 9) Aortic Clamp and Cardioplegia, 10) Anastomoses or Aortotomy, 11) Separation from Bypass, 12) Sternal Closure, and 13) Post-operative Debrief. In the following analysis, the final phase, Post-operative Debrief, was excluded due to large amounts of missing data, resulting in 12 common phases within CABG and AVR procedures.

Within HRV data collected, we are specifically interested in vagally-mediated measures of MWL, namely the root mean square of the successive differences (RMSSD) [20]. Vagally-mediated HRV components have an inverse relationship with the state of MWL they represent, such that higher RMSSD values, measured in milliseconds (ms), indicate lower levels of MWL, and vice versa. According to results from the ELSA-Brasil study, RMSSD values corresponding to the age ranges included in our study should approximate roughly 30 ms under resting conditions [21]. Given the inherently high-demand nature of cardiac surgery, however, values derived from the following analyses are expected to be lower than the reference values obtained through the ELSA-Brasil longitudinal study. HRV was analyzed within each surgical phase and provider role using Kubios HRV software [22], such that one RMSSD value was generated for each role for the duration of each phase.

Statistical analysis using SPSS version 26.0 (Armonk, NY) included a mixed model ANOVA defining surgical phase as a within-subjects factor and provider role as a between-subjects factor. Tukey HSD post hoc comparisons were calculated in response to identification of significant main effects. Results are reported as (mean ± standard deviation, P-value) and are considered significant at the P<0.05 level.

## 3 RESULTS

Primary analysis of physiological data collected from three cardiac surgery team members (attending surgeon, attending anesthesiologist, and primary perfusionist) involved analysis of HRV components from six total cases (N=4 CABG, 2 AVR). Participating clinicians included two surgeons, four anesthesiologists, and three perfusionists. Participant demographics can be found in Table 1

Partial data are missing from various phases within some of these cases due to equipment malfunctioning, which is considered to be unrelated to participant characteristics. Data were transformed to replace missing values using the SPSS method of Linear Trend at a Point, resulting in 18 data points for each phase (3 providers x 6 cases). Using this method, missing values are replaced with their predicted values, based on a regression of the index variable scaled 1 to n [23]. Due to the random nature of the incomplete data within this subset, we do not believe there are specific systematic biases associated with the missing data.

A mixed model ANOVA revealed significant differences (F(22,165)=7.29, p<0.001, *η*p2=0.49) between provider HRV values within surgical phases. Post hoc analysis via Tukey HSD showed that during the Anesthesia Induction phase, anesthesiologists’ (14.36 ± 3.89) MWL was significantly higher than that of the perfusionists (19.13 ± 4.09, P=0.042). During Heparinization, surgeons (11.00 ± 1.65) experienced significantly higher MWL compared to perfusionists (17.56 ± 6.45, P=0.025). The same observation was observed within the Initiation of Bypass phase (surgeons: 9.91 ± 2.72; perfusionists 19.09 ± 7.14, P=0.007), the Aortic Clamp and Cardioplegia phase (surgeon: 9.52 ± 2.91; perfusionist: 18.95 ± 7.04, P=0.007), the Anastomoses or Aortotomy phase (surgeon: 9.45 ± 2.93; perfusionist: 19.00 ± 6.62, P=0.005), the Separation from Bypass phase (surgeon: 10.11 ± 2.26; perfusionist: 17.84 ± 6.32, P=0.026), and the Sternal Closure phase (surgeon: 9.89 ± 3.15; perfusionist: 17.09 ± 7.34, P=0.009). The differences in MWL between surgeons and anesthesiologists during the Aortic Clamp and Cardioplegia phase (surgeon: 9.52 ± 2.91; anesthesiologist: 17.38 ± 4.99, P=0.020) as well as the Anastomoses/Aortotomy phase (surgeon: 9.45 ± 2.93; anesthesiologist: 16.64 ± 4.72, P=0.024) were also significant.

**Figure 1:**
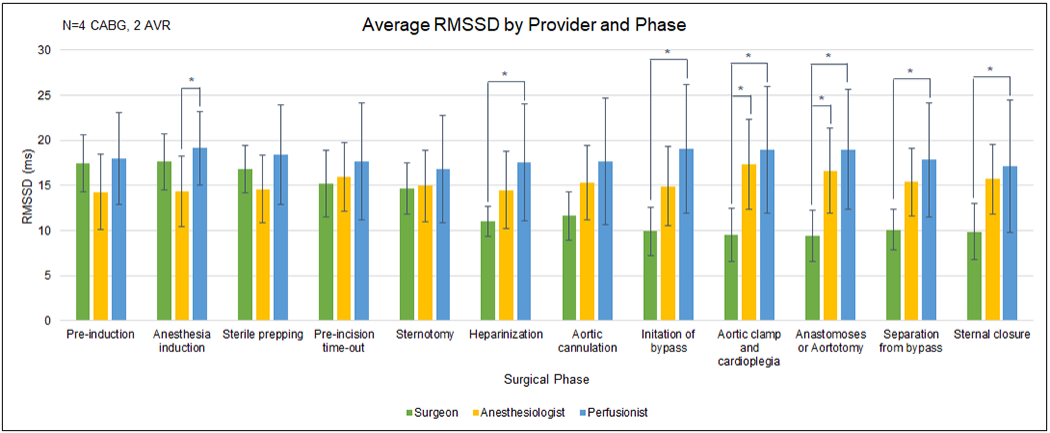
Mental workload (MWL) values via root mean square of the successive differences (RMSSD) for three surgical team members averaged over six total cases. RMSSD, which is lower under high mental demands and higher under low mental demands, was analyzed according to 12 distinct phases common to coronary artery bypass graft (CABG) and aortic valve replacement (AVR) procedures. Between-subjects analysis within each surgical phase reveals significant differences between providers’ MWL during multiple phases of surgery. However, no differences were detected during some of the phases, in particular the pre-incision time-out phase.

Despite significant differences across provider roles during multiple phases of the surgeries, the Pre-incision Time-out phase revealed almost negligible differences across the three roles (range: 2.47 ms), indicating a mirroring of team workload across individuals (Figure 1) while team members engaged in the same task.

## 4 DISCUSSION

Evidence on human factors and teamwork has corroborated that avoidable peri-operative complications might stem from differing perceptions of effective communication, teamwork, and situational awareness by members of the cardiac surgical OR team [24] and that optimal team performance relies on a shared mental model of the team members. A shared mental model can be defined as knowledge by team members that allows them to form correct expectations and coordinate activities among team members [25]. The presence of a shared mental model is suggested to be especially important for teams’ adaptations to change, an ability that is essential in a fast-paced environment of a cardiac OR. Sharing mental models is critical because a team’s capacity to appropriately and dynamically adapt is one of the strongest predictors of team performance.

Recent work by Brown and colleagues identified a lack of shared mental models within and between disciplines caring for cardiac surgical patients across most critical moments during surgery [26]. Brown identified low response variability regarding the importance of specific Pause Points to be a surrogate marker of a shared mental model; in their study, low response variability was detected among all panelists during only 1 of the 12 Pause Points, “immediately before surgical incision.” This finding suggests that there is little agreement between team members over critical moments during cardiac surgery and overall a lack of shared mental models, evident in 11 of 12 identified Pause Points. On the other hand, this finding also highlights the potential inherent importance of pausing to update shared mental models specifically at the moment prior to incision in a coordinated effort to avoid preventable errors [26].

The introduction of objective measures of mental states represents another dimension of rigor to evaluate team dynamics, which has yet to be demonstrated or reported during the pre-incision time-out until now. HRV analysis is the most commonly utilized objective measure of MWL during surgery [14] and primarily consists of time-domain and frequency-domain approaches to analyzing the nature of inter-beat-intervals between heartbeats [27]. Parasympathetically-driven and vagally-mediated measures of HRV, such as RMSSD, are particularly sensitive to temporal fluctuations associated with changing mental demands [28], and this study leverages advancements in technology to lend preliminary support to the sensitivity of HRV in detecting differences in MWL across providers within various surgical phases.

The lack of difference between providers’ HRV within the pre-incision time-out phase may suggest unbiased, objective evidence of shared mental models via psychophysiological mirroring. The lack of difference observed between providers’ HRV in the few remaining phases, including Pre-induction and Sterile Prepping could be reflective of the same mechanisms, but may also be influenced by the relative lack of surgical procedures occurring during these early stages, in combination with greater task engagement of the trainees relative to senior team members. Even during the Sternotomy phase, which marks the beginning of the surgical intervention and similarly revealed no significant differences in HRV across providers, it is often observed that the primary hands-on patient management and interaction is provided by the trainee, while the HRV data acquired through this study captured that of the senior attending team members.

Within these data, HRV values of the cardiac surgery team demonstrate divergent dynamics, evidenced by differences in MWL across provider roles emerging over the course of the surgery. For example, while mirroring of mental states is observed during the pre-incision time-out phase, many remaining downstream phases show significant differences between MWL of at least two roles. During the surgical procedure, almost every phase relies on the three individual team members leveraging specialized skills to complete distinct tasks simultaneously, with a shared common goal held by the team. However, during the pre-incision time-out phase, these dynamics converge, which could be attributed to the team members’ efforts striving to complete not only the same overarching goal, but also the same task. Interleaved episodes of convergence and divergence is indicative of the evolution of adaptive physiological dynamics within the team [29].

Interpretations from this preliminary study are limited by the small sample size and data transformation performed to replace missing data. Nonetheless, the findings observed hold promise for future work in measuring MWL during complex tasks, including surgery “in the wild”. Further, findings may be generalizable to other surgical and invasive settings which may be considered similarly complex.

Future work will use objective HRV measures to investigate teams with high- versus low-convergence in MWL to determine potential determinants of psychophysiological synchrony and/or mirroring. The identification of factors contributing to high- versus low-convergence states may result in targeted training and prevention approaches, advancing the impact of cognitive engineering and human factors approaches to patient safety efforts [1]. Implications of this work include real-time support systems designed to facilitate individual and team-wide situation awareness.

## Figures and Tables

**Table 1:** Participant characteristics of cardiac surgery team members recorded in the cases analyzed.

	Surgeon	Anesthesiologist	Perfusionist
N of Participants	2	4	3
Sex: M (%)	1 (50)	3 (75)	3 (100)
Age: Ave	57	42.25	46
Years of Expereience (Post-Training): Ave	26	9.55	19.33
Cardiac Surgeries Performed (Approx.): Ave	5,500	806	1,933

## CCS CONCEPTS

• **Applied computing** → Life and medical sciences; Health care information systems; Life and medical sciences; Health informatics; Law, social and behavioral sciences; Psychology.

## References

[1] Zenati Marco A, Kennedy-Metz Lauren, and Dias Roger D. 2019. Cognitive Engineering to Improve Patient Safety and Outcomes in Cardiothoracic Surgery. Semin. Thorac. Cardiovasc. Surg 32, 1 (2019), 1–7. DOI:10.1053/j.semtcvs.2019.10.011 31629782 PMC7060831

[2] Joint Commission. 2004. Facts about the Universal Protocol for Preventing Wrong Site, Wrong Procedure, and Wrong Person Surgery.

[3] Gawande Atul and Weiser Thomas. 2009. WHO Guidelines for Safe Surgery 2009: Safe Surgery Saves Lives. DOI:10.1007/978-981-10-5260-6_30 23762968

[4] IOM. 2000. To Err is Human: Building a Safer Health System. The National Academies Press, Washington, D.C. DOI:10.17226/9728 25077248

[5] Russ Stephanie, Rout Shantanu, Sevdalis Nick, Moorthy Krishna, Darzi Ara, and Vincent Charles. 2013. Do safety checklists improve teamwork and communication in the operating room? A systematic review. Ann. Surg 258,6 (2013), 856–871. DOI:10.1097/SLA.0000000000000206 24169160

[6] Patel Janki, Ahmed Kamran, Guru Khurshid A., Khan Fahd, Marsh Howard, Khan Mohammed Shamim, and Dasgupta Prokar. 2014. An overview of the use and implementation of checklists in surgical specialities - A systematic review. Int.J. Surg 12,12 (2014), 1317–1323. DOI:10.1016/j.ijsu.2014.10.031 25448652

[7] Haynes Alex B., Weiser Thomas G., Berry William R., Lipsitz Stuart R., Breizat Abdel-Hadi S., Dellinger E. Patchen, Herbosa Teodoro, Joseph Sudhir, Kibatala Pascience L., Lapitan Marie Carmela M., Merry Alan F., Moorthy Krishna, Reznick Richard K., Taylor Bryce, and Gawande Atul A.. 2009. A Surgical Safety Checklist to Reduce Morbidity and Mortality in a Global Population. N. Engl. J. Med 360, 5 (2009), 491–499. 19144931 10.1056/NEJMsa0810119

[8] Weiser Thomas G., Haynes Alex B., Dziekan Gerald, Berry William R., Lipsitz Stuart R., and Gawande Atul A.. 2010. Effect of A 19-item surgical safety checklist during urgent operations in a global patient population. Ann. Surg 251, 5 (2010), 976–980. DOI:10.1097/SLA.0b013e3181d970e3 20395848

[9] Berrisford Richard G., Wilson Iain H., Davidge Mike, and Sanders David. 2012. Surgical time out checklist with debriefing and multidisciplinary feedback improves venous thromboembolism prophylaxis in thoracic surgery: A prospective audit. Eur. J. Cardio-thoracic Surg 41, 6 (2012), 1326–1329. DOI:10.1093/ejcts/ezr179 22219459

[10] Spanjersberg Alexander J., Ottervanger Jan Paul, Nierich Arno P., Speekenbrink Ron G.H., Stooker Wim, Hoogendoorn Marga, Veghel Dennis van, Houterman Saskia, and Brandon Bravo Bruinsma George J.. 2019. Implementation of a specific Safety Check is associated with lower postoperative mortality in cardiac surgery. J. Thorac. Cardiovasc. Surg (2019). DOI:10.1016/j.jtcvs.2019.07.094 31582206

[11] Fabio Babiloni. 2019.Mental Workload Monitoring: New Perspectives from Neuro-science. In Communications in Computer and Information Science, Longo L and Leva M (eds.). Springer, Cham, 3–19. DOI:10.1007/978-3-030-32423-0

[12] Patel Vimla L., Kannampallil Thomas G., and Shortliffe Edward H.. 2015. Role of cognition in generating and mitigating clinical errors. BMJ Qual. Saf 24, 7 (2015), 468–474. DOI:10.1136/bmjqs-2014-003482 25935928

[13] Suliburk James W., Buck Quentin M., Pirko Chris J., Massarweh Nader N., Barshes Neal R., Singh Hardeep, and Rosengart Todd K.. 2019. Analysis of Human Performance Deficiencies Associated With Surgical Adverse Events. JAMA Netw. Open 2, 7 (2019), e198067. DOI:10.1001/jamanetworkopen.2019.8067 31365107 PMC6669897

[14] Dias Roger D., Ngo-Howard MC, Boskovski MT, Zenati MA, and Yule SJ. 2018. Systematic review of measurement tools to assess surgeons’ intraoperative cognitive workload. Br. J. Surg 105, 5 (2018), 491–501. DOI:10.1002/bjs.10795 29465749 PMC5878696

[15] Durantin G, Gagnon JF, Tremblay S, and Dehais F. 2014. Using near infrared spectroscopy and heart rate variability to detect mental overload. Behav. Brain Res 259, (2014), 16–23. DOI:10.1016/j.bbr.2013.10.042 24184083

[16] Gao Qin, Wang Yang, Song Fei, Li Zhizhong, and Dong Xiaolu. 2013. Mental workload measurement for emergency operating procedures in digital nuclear power plants. Ergonomics 56, 7 (2013), 1070–1085. DOI:10.1080/00140139.2013.790483 23654299

[17] Zenati Marco A., Leissner Kay, Zorca Suzana, Kennedy-Metz Lauren, Yule S, and Dias Roger D.. 2019. First Reported Use of Team Cognitive Workload for Root Cause Analysis in Cardiac Surgery. Semin. Thorac. Cardiovasc. Surg 31, 3 (2019), 394–396. DOI:10.1053/J.SEMTCVS.2018.12.003 30578828 PMC6584063

[18] Kennedy-Metz Lauren R., Dias Roger D., Stevens Ronald H., Yule Steven J., and Zenati Marco A.. Analysis of Mirrored Psychophysiological Change of Cardiac Surgery Team Members During Open Surgery. J. Surg. Educ. (2020), 1–8. 10.1016/j.jsurg.2020.08.012. PMC7904574 32863172

[19] Conboy Heather M., Avrunin George S., Clarke Lori A., Osterweil Leon J., Goldman Julian M., Yule S, Zenati Marco A., and Christov Stefan C.. 2017. Cognitive support during high-consequence episodes of care in cardiovascular surgery. 2017 IEEE Conf. Cogn. Comput. Asp. Situat. Manag. CogSIMA 2017 (2017). DOI:10.1109/COGSIMA.2017.7929610 PMC5526347 28752132

[20] Shaffer Fred and Ginsberg JP. 2017. An Overview of Heart Rate Variability Metrics and Norms. Front. Public Heal 5, 9 (2017), 1–17. DOI:10.3389/fpubh.2017.00258 PMC5624990 29034226

[21] Dantas Eduardo Miranda, Kemp Andrew Haddon, Andreão Rodrigo Varejão, Dias da Silva Valdo José, Brunoni André Russowsky, Hoshi Rosangela Akemi, Bensenor Isabela Martins, Lotufo Paulo Andrade, Pinho Ribeiro Antonio Luiz, and Mill José Geraldo. 2018. Reference values for short-term resting-state heart rate variability in healthy adults: Results from the Brazilian Longitudinal Study of Adult Health—ELSA-Brasil study. Psychophysiology 55, 6 (2018). DOI:10.1111/psyp.13052 29292837

[22] Tarvainen Mika P., Niskanen Juha Pekka, Lipponen Jukka A., Rantaaho Perttu O., and Karjalainen Pasi A.. 2014. Kubios HRV - Heart rate variability analysis software. Comput. Methods Programs Biomed. 113, 1 (2014), 210–220. DOI:10.1016/j.cmpb.2013.07.024 24054542

[23] Cokluk Omay and Kayri Murat. 2011. The effects of methods of imputation for missing values on the validity and reliability of scales. Kuram ve Uygulamada Egit. Bilim 11, 1 (2011), 303–309.

[24] Lim Beng-chong and Klein Katherine J. 2006. Team mental models and team performance: A field study of the effects of team mental model similarity and accuracy. J. Organ. Behav 27, (2006), 403–418.

[25] Cannon-Bowers Janis A., Salas Eduardo, and Converse SA. 1990. Cognitive psychology and team training: Training shared mental models of complex systems. Hum. Factors Soc. Bull 33, 12 (1990), 1–4.

[26] Brown Evans K.H., Harder Kathleen A., Apostolidou Ioanna, Wahr Joyce A., Shook Douglas C., Farivar R. Saeid, Perry Tjorvi E., and Konia Mojca R.. 2017. Identifying variability in mental models within and between disciplines caring for the cardiac surgical patient. Anesth. Analg 125, 1 (2017), 29–37. DOI:10.1213/ANE.0000000000002087 28537973

[27] Task Force of the European Society of Cardiology and the North American Society of Pacing and Electrophysiology. 1996. Heart rate variability: Standards of measurement, physiological interpretation and clinical use. Eur. Heart J 17, (1996), 354–381. DOI:10.1161/01.CIR.93.5.1043 8737210

[28] Shaffer Fred, McCraty Rollin, and Zerr Christopher L. 2014. A healthy heart is not a metronome: An integrative review of the heart’s anatomy and heart rate variability. Front. Psychol. 5, 9 (2014), 1–19. DOI:10.3389/fpsyg.2014.01040 25324790 PMC4179748

[29] Kazi Sadaf, Khaleghzadegan Salar, Dinh Julie, Shelhamer Mark, Sapirstein Adam, Goeddel Lee, Chime Nnenna, Salas Eduardo, and Rosen Michael. 2019. Team Physiological Dynamics: A Critical Review. Hum. Factors (2019). DOI:10.1177/0018720819874160 31557057

